# Immunomodulatory and Antiaging Mechanisms of Resveratrol, Rapamycin, and Metformin: Focus on mTOR and AMPK Signaling Networks

**DOI:** 10.3390/ph15080912

**Published:** 2022-07-23

**Authors:** Vincenzo Sorrenti, Francesca Benedetti, Alessandro Buriani, Stefano Fortinguerra, Giada Caudullo, Sergio Davinelli, Davide Zella, Giovanni Scapagnini

**Affiliations:** 1 Department of Pharmaceutical and Pharmacological Sciences, University of Padua, Largo Egidio Meneghetti, 2, 35131 Padova, Italy; 2 Bendessere® Study Center, Via Prima Strada 23/3, 35129 Padova, Italy; giada.caudullo@solgar.it; 3 Maria Paola Belloni Center for Personalized Medicine, Data Medica Group (Synlab Limited), 35100 Padova, Italy; 4 Department of Biochemistry and Molecular Biology, Institute of Human Virology, University of Maryland School of Medicine, Baltimore, MD 21201, USA; fbenedetti@ihv.umaryland.edu (F.B.); alessandro.buriani@gmail.com (A.B.); 5 IRCCS SDN—Via E. Gianturco 113, 80143 Napoli, Italy; stefano.fortinguerra@gmail.com; 6 Department of Medicine and Health Sciences “V. Tiberio”, University of Molise, 86100 Campobasso, Italy; sergio.davinelli@unimol.it

**Keywords:** resveratrol, rapamycin, metformin, AMPK, mTOR, immunosenescence, aging, immunomodulators, signaling pathways, immunity

## Abstract

Aging results from the progressive dysregulation of several molecular pathways and mTOR and AMPK signaling have been suggested to play a role in the complex changes in key biological networks involved in cellular senescence. Moreover, multiple factors, including poor nutritional balance, drive immunosenescence progression, one of the meaningful aspects of aging. Unsurprisingly, nutraceutical and pharmacological interventions could help maintain an optimal biological response by providing essential bioactive micronutrients required for the development, maintenance, and the expression of the immune response at all stages of life. In this regard, many studies have provided evidence of potential antiaging properties of resveratrol, as well as rapamycin and metformin. Indeed, in vitro and in vivo models have demonstrated for these molecules a number of positive effects associated with healthy aging. The current review focuses on the mechanisms of action of these three important compounds and their suggested use for the clinical treatment of immunosenescence and aging.

## 1. Immune Changes in Immunosenescence and Aging

Aging is a highly complex and multilayered process, and to understand it, we require a holistic approach. Indeed, the aging process is a phenomenon resulting from the dysregulation of several complex systems that are interdependent and mutually interacting [1], and a relevant change occurring with aging is linked to the remodeling of the architecture and functioning of the immune system. This progressive change leads to reduced immune efficacy, hampered immune response to vaccination, resulting in enhanced vulnerability to infectious diseases, and increased risk of age-related chronic immune-inflammatory diseases [2,3]. This phenomenon, which Roy Walford named “immunosenescence”, has a marked influence on naïve and acquired immunity, dramatically affecting T lymphocytes [4,5].

Multiple factors, such as genetics, exercise, nutrition, biological triggers, and early exposure to certain microorganisms, are reported to favor immunosenescence [6]. In fact, one of the major age-related changes is the dysregulation of immune signaling around puberty when, due to thymic involution, this developmentally programmed event occurs. Consequently, the adaptive immune system phenotypically changes at the cellular level and cell subset distribution, resulting in functional alterations. At the same time, innate immunity is also affected [7,8]. In this regard, a critical age-associated immune alteration is the reduction in peripheral blood naïve cell number and the induction of memory cells [9], which results in a progressive decrease in cellular responses and antibody production against various pathogens. These changes are also accompanied by the low-grade inflammation present commonly in older adults, a phenomenon nicknamed inflammaging [10]. Additional senescence hallmarks appear, such as alterations in telomere length, impaired mitochondrial function, and altered response to xenobiotics [11].

However, according to a competing theory, immune changes concomitant with aging could be seen as an adaptive physiological response, so it remains an open question as to whether they should be reversed or just controlled, taking into account that such changes may lead to health issues in the long term [11,12].

### 1.1. Innate Immune Response during Aging

**Figure 1:**
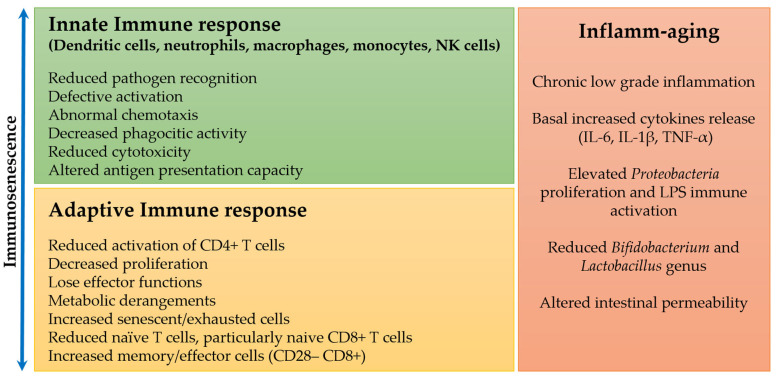
Changes in the innate and adaptive immune response and chronic inflammation during immunosenescence and aging.

The innate immune response represents a well-preserved phylogenetically first line of defense against pathogens of various types, and during aging, this type of immune response appears to be less involved than the adaptive one in the immune changes, though some remarkable alterations have been highlighted [13,14] (Figure 1).

It has been shown that the adhesive capacity and phagocytic activity of neutrophils and macrophages are not different in the elderly compared to young subjects [15,16]. On the other hand, their chemotaxis and production of free radicals and cytokines appear to decrease with age [16,17,18], but these data need to be confirmed by additional studies involving human subjects. Additionally, natural killer (NK) cells have a reduced cytotoxic function at the single-cell level, which is, however, compensated by an expansion in their number to maintain or even enhance whole functionality [19,20].

Of great significance in elderly subjects are the changes in altered antigen presentation capacity, particularly of differentiated dendritic cells (DCs), and consequently reduced activation of CD4^+^ T cells, with no significant difference in cell number. Whether this phenomenon is related to the decreased ability to present the antigen or inability to recognize it remains unclear. Interestingly, these DCs secrete a high quantity of proinflammatory cytokines even when they are in the quiescent state, as determined by the expression of immune receptors such as Fc and C3b receptors, or Toll-like receptors (TLRs), indicating a basal activity that could characterize the higher degree of chronic inflammation observed in some older adults, compared to younger subjects [21,22]. Further studies are ongoing to determine the cause of the chronic activation of these DCs [23,24].

### 1.2. Adaptive Immune Response during Aging

The adaptive immune response exhibits numerous and substantial phenotypic changes with aging, in particular in CD8^+^ T cells and to a lesser extent in CD4^+^ T cells. Circulating naive CD8^+^ T cells decrease in number and frequency, while the opposite is true for memory cells (CD28^–^ CD8^+^) and differentiated effector T cells (CD45RA^+^ CD28^–^ CD8^+^). This change is mainly due to chronic, lifelong antigenic exposures from pathogenic sources and/or intrinsic stresses such as oxidative stress, tissue damage, and inflammation, which can modify self-antigens [9,25].

Herpes viruses (HSV) represent some of the main pathogens that alter the immune response during aging. In particular, the adaptive immune response in its T-cell components appears to be more severely affected by cytomegalovirus (CMV) infections. The pioneering Swedish immune longitudinal studies OCTO and NONA, realized in elderly Swedish subjects, for 11 years, have identified an immune risk phenotype (IRP) linked to higher mortality during the follow-up period. The results of two studies indicated that the combination of higher CD3^+^ CD8^+^, decreased CD3^+^ CD4^+^, and poor proliferative response to mitogenic stimulation was associated with an increase in mortality in a 2-year follow-up. These changes were significantly associated with seropositive responses to CMV [26,27,28,29]. However, recent evidence puts the CMV infection into question as the main cause of immunosenescence during aging, as it can be seen as a continuous stimulation maintaining sustained immunological alertness and promoting a better immune response [3,30]. Aligned with this view is the evidence of the functional accumulation of senescent memory T cells. According to Hayflick’s replicative senescence model, these cells cannot replicate while remaining dysregulated though metabolically active, thus secreting several inflammatory cytokines, chemokines, growth factors, and matrix remodeling factors, known as senescence-associated secretory phenotype (SASP), altering the local tissue environment and contributing to chronic inflammation [31,32]. However, when CMV is reactivated, these cells are still able to function. It is thus reasonable to believe that senescent immune cells are not only harmful for specific cellular functions but also useful in other physiological processes, such as tissue repairing and fighting cancerous transformation [33]. Consequently, it is particularly important to identify molecular markers that allow discrimination between senescent and exhausted cells. Considering that senescent immune cells may be functionally “dormant”, while exhausted immune cells may be functionally inert, this distinction would become crucial when analyzing immune functions in relation to aging [34]. Finally, it has been observed that senescent T lymphocytes can be “awakened” by the engagement of certain surface receptors (e.g., CTLA-4, PD-1, TIM-3LAG-3, and TIMIN) called immune checkpoint inhibitors, which can lead to reactivation [3].

## 2. Key Signaling Pathways Involved in Immunosenescence: Focus on AMPK and mTOR

Lymphocyte subpopulations can be altered in most immune-related diseases, and their activation or modulation can contribute to the progression or resolution of disease conditions. Moreover, depending on the sets of cytokines and growth factors they produce, lymphocytes and other leukocytes can be phenotypically defined as effectors or regulators and accordingly play their role in the complex immune-inflammatory chain of events leading to amplification, suppression, or chronicization of the immune response [35]. In addition, the ability of lymphocytes and other immune cells to amplify or restrict each other’s activity mostly via the cytokine-mediated crosstalk generates a network phenomenon potentially able to reshape cellular phenotypes and orient the immune equilibrium towards different homeostatic balances, possibly leading to chronic inflammatory conditions. In this regard, we note that the aging process provides an example of the progressive dysregulation of an immunosuppressive network, where regulatory immune cells are increased, including the regulatory subtypes of T (Treg) and B (Breg) lymphocytes, and regulatory natural killer cells (NKreg), macrophages (Mreg), dendritic cells (DCreg), and inflammatory mediator-induced suppressor immature myeloid cells. Indeed, lymphocytes and other immune cells participating in the immunosuppressive pathways share the same signaling molecules, enhancing the expression of suppressive genes and related proteins. In addition, it has been suggested that this co-operative immunosuppressive network is stimulated in inflammaging, the chronic low-grade inflammatory condition associated with immunosenescence, where the remodeling of the immune system leads to the reduced effectiveness of the immune response and its functional decline with aging [36,37].

Several cellular molecular mechanisms have been proposed to better explain the immunosenescence process, to identify therapeutic strategies to improve the immune response in the elderly and chronic conditions. In this regard, some strategies have been developed to target the main immune effectors, T lymphocytes. These strategies rely on the notion that, at the molecular level, the transcription factor T-cell factor 1 (TCF1), a direct actor of the WNT/β-catenin signaling pathway, controls common gene-regulatory networks and is indispensable for T-cell development and T-cell responses. TCF1 appears to be pivotal in the development of effector and memory precursor and stem-like T cells, following T lymphocyte stimulation and division. TCF1 expression declines in aging, and naïve and memory T cells from older adults have decreased TCF1 expression compared to young ones. In addition, TCF1, in concert with the transcription factor Yin Yang-1 (YY1), acts as a transcriptional activator of pri-miR-181a, whose expression in naïve CD4 and CD8 T cells also decreases with age. In particular, proliferating old T cells have the prolonged activation of the AKT-mTOR pathway, which results in the sustained repression of FOXO1 and consequently reduced TCF1 expression, which favors aged T-cell differentiation into TCF1 low terminal (short-lived) effector cells at the expense of memory precursors cells, thus progressively declining the effectiveness of the immune response [38]. A better understanding of key molecular pathways involved in immunosenescence is thus particularly relevant to identify synthetic and natural molecules, possibly acting as antiaging agents or geroprotectors at key molecular steps.

One of the most important cellular pathways involves the AMP-activated protein kinase (AMPK), and it plays a crucial part in tissue energy regulation and in immune response by working together with immune signaling pathways involved in innate and adaptive immunity, thus influencing immunometabolism and the functions of immune cells [3,39]. AMPK activation controls cellular immunity along with immune signaling pathways and modulates energy metabolism, which subsequently affects immune cell activation. Indeed, AMPK activity can drive many functions of innate and adaptive immunity by controlling immune cell differentiation and activity. In particular the activation of the AMPK signaling pathway negatively modulates the activity of the NF-κB (nuclear factor-κB) network, resulting in the suppression of the proinflammatory responses [40]. It is therefore evident that AMPK regulation is an intricate, context-related process that can paradoxically exert opposite effects in chronic diseases. To this regard, AMPK activation inhibits various relevant immune signaling pathways, for example, the NF-κB, JAK/STAT, C/EBPβ, HIF-1α and CHOP pathways. Moreover, besides NF-κB inhibition, the activation of AMPK can suppress inflammatory responses by inhibiting the signal transducer and activation pathways of transcription (STAT). All these data indicate that when AMPK function is altered in many chronic diseases, cellular homeostasis may be negatively affected [2,41].

**Figure 2:**
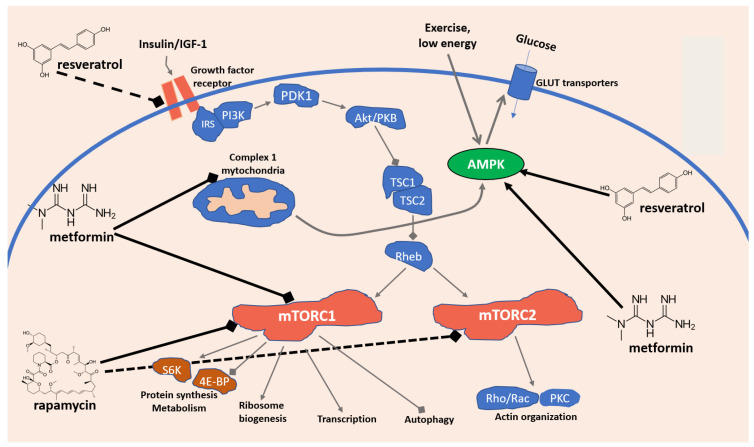
Simplified model of the mTOR signaling network in mammalian cells and antiaging mechanisms of resveratrol, metformin, and rapamycin.

Another important pathway involves the mechanistic target of rapamycin (mTOR), a checkpoint protein Ser/Thr kinase which receives and integrates inputs from its upstream regulators, the energy status sensitive AMPK pathway, the insulin/IGF–PI3K (growth factor) signaling pathway, as well as their shared nutrients and stress-sensitive TSC1–TSC2 complex and Rheb, and in turn controls a number of processes, including cell growth, transcription, translation, ribosome formation, molecular transport, and autophagy. Such key regulatory role in cellular life has long suggested mTOR as one of the most important regulators of the cellular lifespan and aging. In particular, in mammalians, mTOR exists in two forms, mTOR1 and mTOR2, that together with other proteins (raptor and mLST8 for mTOR1, and rictor and mLST8 for mTOR2) form two different molecular complexes: the Rapamycin-sensitive mTOR Complex 1 (mTORC1) and the Rapamycin-insensitive mTOR Complex 2 (mTORC2). The first one oversees time and the modality of cell growth, while the second one regulates the spatial arrangements of the cell growth. While during development TOR primarily regulates growth, in the adult, when growth is reduced, TOR regulates aging and other facets of nutrient-related functions (Figure 2) [42,43,44].

The central role of the mTOR-dependent pathway has been highlighted in several contexts, including immunosenescence, a phenomenon affecting both innate and adaptive immunity during aging, as well as inflammaging, the low-grade inflammatory state, both associated in the elderly with an increased innate immune susceptibility and cytokine release. In addition, the increase in dysfunctional immunosenescent cells, the chronic persistence of cytokine unbalance, and the consequent remodeling and rebalancing of immune cells, coupled with the failure to effectively trigger the adaptive immune response, have been suggested to explain why the elderly seem to be more susceptible to SARS-CoV-2 infection and more frequently respond with an abnormal increase in inflammatory mediators, leading to a life-threatening cytokine storm, endothelial injury, and disseminated organ injury [45].

Further highlighting its importance, a study associating markers of immunosenescence with specific molecular pathways using genomics, proteomics, and a system biology approach, confirmed an association between mTOR and immunosenescence. In particular transcriptomic, proteomic, and DNA methylation data informed the generation of models for key molecular pathways-associated networks and their correlation with immunosenescent markers. This, in turn, provided a better understanding of the effect of immunosenescence on biological networks of immune response. Indeed, various sets of biological pathways and functional gene sets were demonstrated to have a significant association with immunosenescence processes, among which the mTOR signaling pathway [46]. In addition, in another study, the mTOR inhibitor RAD001 was utilized to indirectly evaluate the effect of mTOR modulation on immunosenescence, by measuring the ability to improve vaccination response in elderly volunteers. The results indicated a significant enhancement of the response to the influenza vaccine. Although the study did not analyze parameters directly related to mTOR, the result strongly suggests its association with immunosenescence in humans [47]. Finally, immunosenescence and inflammaging in older adults have been suggested to affect immune surveillance and predispose them to SARS-CoV-2 infection and serious COVID-19 complications. On this basis, and considering its known effect on age-related diseases, the mTOR inhibitor rapamycin (sirolimus) is presently being tested in clinical trials to evaluate its effectiveness against COVID-19 [48,49].

## 3. Dietary and Nutraceutical Strategies to Tackle Immunosenescence and Aging: Focus on Resveratrol, Rapamycin, and Metformin

Poor nutritional status during aging represents a crucial element in driving immunosenescence progression. Consequently, proper dietary interventions are believed to help maintain an optimal immune response by providing bioactive micronutrients (for example, minerals and vitamins such as zinc, vitamin C, D, and B12) required for the development, maintenance, and expression of the immune response at all stages of life. Unsurprisingly, the deficiency of some of these micronutrients compromises the proper immune response, increasing susceptibility to infections. Moreover, several other compounds, such as omega 3 fatty acids, specific amino acids, phytochemicals (e.g., resveratrol and astaxanthin), and natural-derived drugs such as rapamycin and metformin, are known to exert antiaging, antiviral, and immunomodulatory effects and maintain a healthy immune system [2,50,51,52,53]. In particular, polyphenols such as curcumin, flavanols, and resveratrol interact with the gut microbiota where they undergo extensive metabolism to produce small molecules active on transcription factors (Nrf2, PGC1-α, FoXO3, AMPK, Sirt1) involved in several cellular functions, including mitochondrial biogenesis, antioxidant systems, glucose and lipid homeostasis, DNA repair, and immune homeostasis [54,55,56,57]. In a recent review, our group discussed the antiviral role of resveratrol, rapamycin, and metformin which is strictly related to immunosenescence and aging [58]. In this review, we will be focusing on resveratrol, rapamycin, and metformin with regard to their immunomodulatory and antiaging effects.

### 3.1. Resveratrol

Resveratrol (3,5,4′-trihydroxy-stilbene) is a natural phytoalexin (from the Greek *aléxein* = to guard or protect), produced as a stress-signaling molecule by plants, such as *Polygonum cuspidatum*, in response to nutrient deficiency and environmental changes, both to protect against dangerous situations (UV rays, pathogens, etc.) and to provide early defensive interventions to survive. Two resveratrol isomers, the *trans* or *cis* configuration of phenolic rings, can exist in nature. The *trans* form appears to be the most stable and biologically active. Resveratrol can be extracted from different sources, including grapes, peanuts, red wine, a variety of berries, and some medicinal phytocomplexes [59,60]. Despite its lipophilic nature that makes it easily absorbed, metabolized, and excreted, resveratrol possesses a short half-life that limits its use [61,62,63], and the type of food ingested, as well as the way it is consumed, influences its absorption [64]. Following oral administration, resveratrol forms complexes with membrane transporters, favoring its absorption by passive diffusion, and diffusion in the bloodstream, where it can be detected in three main forms: glucuronide, sulfate, and free [64]. The latter is typically bound to albumin or lipoproteins, allowing a better distribution and entry into the cells [65]. Resveratrol metabolism mainly occurs in the liver, producing a plethora of metabolites detectable in urines [66]. Given its many biologic activities, resveratrol has been proposed as a dietary supplement for human consumption useful in immune disorders. However, pharmacokinetic analysis reveals a rapid metabolism of resveratrol. Its half-life after oral administration is very low, despite absorption reaching 70%. This impacts the physiological significance of the high concentrations used in in vitro studies [62].

Many studies have provided evidence of neuroprotective, antiatherogenic, antithrombotic, anti-hypercholesterolemic, vasorelaxant, and anticancer properties of resveratrol. Both in vitro and in vivo studies showed positive health outcomes with the use of resveratrol [67,68,69,70], including the reduction of inflammation [71] and anticancer [72,73,74,75,76], antioxidant, and antiaging properties [77,78,79]. In addition, resveratrol extends the lifespan of different evolutionarily distant species, including *Caenorhabditis* *elegans* [80], *Saccharomyces* *cerevisiae* [81], and *Drosophila melanogaster* [82], highlighting the conserved nature of the involved pathways. For this reason, resveratrol represents a promising compound to be used for the prevention and treatment of cardiovascular and neurodegenerative autoimmune disorders, as well as some chronic diseases [83,84,85,86,87].

#### Resveratrol as an Immunomodulator

Resveratrol can exhibit antioxidative, anticancer, antimicrobial, anti-neurodegenerative, and estrogenic properties. In addition, resveratrol also has an immunomodulatory/anti-inflammatory role, which includes the inhibition of spleen cell proliferation induced by concanavalin A (ConA), IL-2, or alloantigens, and more efficiently prevents the production of IL-2 and IFNγ by lymphocytes and the production of tumor necrosis factor-alpha (TNF-α) or IL-12 by macrophages (see Box 1) [88]. Moreover, by interacting with several molecular targets, resveratrol regulates innate and adaptive immunity. Nevertheless, in a dose-dependent manner, resveratrol modulates immune functions; at low doses, it stimulates the immune system, whereas at high doses, it can induce immunosuppression. In rodent animal models, resveratrol reduced inflammatory responses in peritonitis, improved the immune response against cancer cells, and reversed immunosenescence in older rats [89]. Moreover, resveratrol has been found to activate macrophages, T cells, and natural killer cells (NK), and can regulate CD4^+^CD25^+^ regulatory T-cell suppressive functions [89]. The reduction in reactive oxygen species (ROS), the inhibition of cyclooxygenase (COX), and the activation of many anti-inflammatory pathways, including the one controlled by Sirtuin-1 (Sirt1) are the main resveratrol antiaging mechanisms of action [90]. In particular, Sirt1 negatively interferes with the TLR4/NF-κB/STAT signal, which in turn reduces cytokine secretion from inactivated immune cells and modulates macrophage/mast cell-derived proinflammatory factors, such as histamine and platelet-activating factor (PAF), TNF-α [91]. Sirt1 activation by resveratrol can also deacetylate the transcription factor STAT3, interfering with nuclear factors relevant for th17 lymphocyte differentiation [92]. In a recent review by Chhabra et al., the antitumor effects of resveratrol have been correlated with its ability to enhance antitumor immunity and reverse the immunosuppressive tumor microenvironment [93]. In particular, resveratrol exerts an immunomodulating mechanism by restoring T-cell function, targeting the immune-checkpoint signaling PD-1/PD-L1. Furthermore, resveratrol can activate NK cells through AKT- and mTORC2-mediated c-Myb upregulation [93].

Box 1 **Immune-mediated mechanisms of resveratrol, rapamycin, and metformin.**

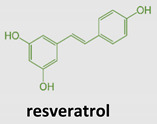



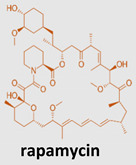



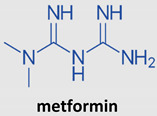


⮚
**Immunosuppressant at high concentration and immunomodulator at low/normal concentration**

✓Negatively regulates IL-2 and IFNγ by lymphocytes and TNF-α and IL-12 by macrophages✓Reduced immune-inflammatory responses✓Activates Sirt1 which negatively interferes with the TLR4/NF-κB/STAT signal, with reduced cytokines secretion from inactivated immune cells and macrophage/mast cell-derived pro-inflammatory factors.✓Activates macrophages, T cells, and NK cells✓Regulates CD4+CD25+ regulatory T cell suppressive functions✓Activates Sirt1 which deacetylates STAT3 interfering with th17 lymphocyte differentiation✓Restores T-cell function targeting the immune-checkpoint signaling PD-1/PD-L1✓Activates NK cells through AKT- and mTORC2-mediated c-Myb upregulation
⮚
**Mostly immunosuppressant but immunomodulator in the elderly**

✓Provides general inhibitory effects on the immune system✓Ameliorates some immune functions in the elderly, response to vaccination and improved outcomes from infections✓Inactivates T and B lymphocytes✓Impairs lipid to glucose metabolism and immune suppression in long-term use✓Extends lifespan and delay aging in model organisms✓Reduces p16INK4A protein expression and increases collagen VII protein levels in the skin✓Reduces the negative effects on the immune system in COVID-19 patients
⮚
**ostly immunomodulator**

✓Exerts direct immunomodulatory effect on immune cells by AMPK induction and mTORC1 inhibition✓Promotes health immunity via an H3K4 methyltransferase/demethylase complex, downregulating some targets, including mTOR and S6 kinase✓Increases the differentiation of naïve T cells into both regulatory and memory T cells, while decreasing the ability of neutrophils to initiate in NETosis.✓Exerts immunomodulatory effects and contributes to the reduction of lung metastases of melanoma cell✓Increases the number and function of tumor-infiltrating lymphocytes (TILs) and inhibits oxidative phosphorylation in B cells✓Reduces the stability and localization of PD-L1✓Contributes to the improvement of CTLs against cancer cells✓Reduces the burden of autoimmune diseases in several animal models and some human studies

Future studies to uncover the resveratrol immunomodulatory mechanism or with new resveratrol analogs and derivatives, exerting superior immunomodulatory properties, or a combination of resveratrol with other compounds should be performed together with clinical trials.

### 3.2. Rapamycin

Rapamycin, also known as sirolimus, is produced by bacteria to inhibit fungal growth and it is an interesting pharmacologically active macrolide from drug-rich streptomyces bacteria, carrying an exotic name from the Easter Island origins of the *Streptomyces hygroscopicus* sample that produces it [94]. However, its development as an antifungal was abandoned when it was discovered to have potent immunosuppressive and antiproliferative properties due to its ability to inhibit the pleiotropic molecular regulator mTOR (mammalian target of rapamycin). mTOR is a Serine/Threonine (Ser/Thr) protein kinase, of the phosphatidylinositol-3-kinase-related kinase (PIKK) family acting as a central controller regulating the processes of cell growth, survival, metabolism, proliferation, and autophagy.

By inhibiting the mTOR signaling pathway, rapamycin can exert a diverse spectrum of pharmacological activities ranging from antiproliferative to anti-inflammatory to the regulation of autophagy and apoptosis [43,95]. Actually, many more biological effects have been identified or hypothesized for its application in a wide spectrum of diseases, from cancer to autoimmune diseases, hypertension, osteoporosis, diabetes, and neurodegenerative conditions such as Alzheimer’s and Parkinson’s diseases [96]. Indeed rapamycin and its analogs have been demonstrated in vitro and in vivo to slow aging, extend lifespan, and prevent age-related diseases, including diabetic complications such as retinopathy, and have been approved by the FDA for the treatment of several conditions [97].

#### Rapamycin as an Immunomodulator

Rapamycin is an mTOR inhibitor, and thus affects the immune system, most significantly leading to the inactivation of T and B lymphocytes by reducing their response to IL-2 and other cytokine receptor-dependent signal transduction mechanisms (see Box 1) [98,99,100,101,102]. Consequently, rapamycin has mostly been developed as an immunosuppressant. For example, thanks to its mTOR inhibitory effects on the immune system, sirolimus is reported to be effective and well-tolerated in the treatment of systemic lupus erythematosus [103,104], as well as in patients with primary relapsed/refractory autoimmune cytopenia [105]. Most significantly, sirolimus is a pharmacological tool suitable for organ transplant rejection, representing its primary field of clinical application. In fact, sirolimus has been approved by the FDA and is used for the prophylaxis of organ rejection in patients aged 13 years or older receiving renal transplants [106].

A growing number of aging-related conditions have been suggested to benefit from mTOR inhibition, including among others, age-related blindness, rheumatoid arthritis, osteoporosis, cardiovascular diseases, muscular dystrophy, diabetic nephropathy, age-related cancer, neurodegeneration, and adult polycystic kidney disease [107]. In addition, among the key cellular functions and processes regulated by mTOR, cell growth, development, and senescence have attracted interest in fields such as cancer and aging research. In particular, mTOR has been associated with an increased lifespan by modulating the processes of transcription, translation, autophagy, and metabolism [108]. mTOR was initially shown to be important in reducing aging effects with the observation that its inhibitor rapamycin could extend lifespan and delay aging in model organisms, including a murine in vivo model of mTOR inhibition [109]. The applicability of chronic mTOR attenuation in aging-associated diseases such as cerebrovascular dysfunction and cognitive decline has also been addressed, and significant benefits were shown in experimental animal models of cerebrovascular and neuronal dysfunction, thus suggesting that mTOR inhibitors such as rapamycin may decrease the risk of developing age-associated neurological disorders including vascular dementia and Alzheimer’s disease [110]. The possibility of the development of pharmacological antiaging interventions with rapamycin and its derivatives (rapalogs) has thus attracted extensive research. However, this approach has been hampered by a set of side effects associated with long-term rapamycin administration, a regimen necessary for any antiaging treatment, ranging from impaired lipid to glucose metabolism and immune suppression. mTOR forms two different protein complexes, mTORC1, which is acutely sensitive to rapamycin, and mTORC2, which is only sensitive to rapamycin when it is administered chronically in vivo. For this reason, the side effects seen with the chronic administration of rapamycin have been attributed to the inhibition of mTORC2, thus prompting the quest for mTORC1 specific analogs or the development of alternative treatment regimens aiming to selectively inhibit mTORC1, such as intermittent rapamycin administration [109,111,112,113]. Recently, there have been interesting clinical results regarding the effect of rapamycin on the aging of the skin. An exploratory, placebo-controlled, interventional trial was conducted where rapamycin was administered topically to participants older than 40 years, with evidence of age-related photoaging and dermal volume loss. When markers of aging were evaluated, it was observed that topical rapamycin application significantly reduced p16INK4A protein expression and increased collagen VII protein levels in the skin of the treated subjects, a finding consistent with a reduction in cellular senescence, which was also confirmed by the relative improvement in the clinical appearance of the skin, thus indicating the effectiveness of the treatment [114].

Finally, we note that immunosenescence is an area of growing interest in the pharmacology of aging. Given their immunosuppressant effect, the use of rapamycin and rapalogs for geroprotective strategies in immunosenescence may appear counterintuitive. However, growing evidence suggests that mTOR inhibition by rapamycin ameliorates some immune functions in the elderly, including response to vaccination and improved outcomes from infections [115,116,117]. In this regard, in 2006, an explanation of this apparent biological paradox was proposed, considering senescence as a quasi-program of cellular post-development [96]. According to this theory, once the initial development is complete, it does not totally switch off and continues as a quasi-program, leading slowly to a secondary decline, when senescent cells acquire phenotypic changes that contribute to aging and age-related diseases. Cell senescence is thus associated with the activation of mTOR. This could explain how its pharmacological inhibition could then reverse senescence and its deleterious consequences on the immune system [96]. Geroprotective strategies using rapamycin and rapalogs such as everolimus have been proposed to tackle immunosenescence, especially in therapeutic strategies to fight infectious diseases caused by pathogens that are more infectious and prevalent in the elderly [115], gerophylic, or with more severe symptoms and lethality, and gerolavic, such as SARS-CoV-2, the etiologic agent of COVID-19. The reduced immune functions due to immunosenescence have indeed been suggested as one of the possible causes of the age-associated increase in the COVID-19 infection rate, severity, and lethality. Consequently, experimental geroprotective strategies using everolimus and sirolimus alone or in combination with other molecules have been recently proposed to treat some of the negative effects on the immune system in COVID-19 patients [118,119,120,121].

### 3.3. Metformin

Metformin, a biguanide antidiabetic drug, has been receiving attention thanks to its anti-inflammatory and immunomodulatory properties [122]. Metformin, a drug derived from Galega officinalis, reduces blood glucose levels by suppressing hepatic glucose production in patients with type 2 diabetes [123,124,125]. When used in some in vivo models, metformin administration increased the lifespan of *Caenorhabditis elegans* [125,126,127] and mice [128,129]. Furthermore, metformin has been reported to benefit patients with cardiovascular diseases [130] and treat metabolic syndromes and cancer and in the prevention and treatment of aging and aging-related diseases [131,132]. 

The mechanisms of action of metformin in recent years have been extensively studied [133,134,135]. At the molecular level, many targets have been identified [136,137], mostly focusing on the respiratory complex I of the electron transport chain [138,139]. The observed inhibition of complex I has been mainly attributed to ATP deficiency, with reduced oxidative phosphorylation and direct effects on ATP-requiring reactions and indirect effects on AMPK activation [134,137,138]. Indeed, there are many downstream consequences of AMPK activation, such as the inhibition of fatty acid synthesis and the mechanistic target of the rapamycin signaling network (mTOR) [140], both of which lead to reduced energy consumption. More recently, it has been recognized that the inhibition of complex I has further consequences related to the accumulation of NADH than NAD+, influencing cellular biochemistry [141]. In this regard, metformin inhibits NADH-ubiquinone oxidoreductase on the mitochondrial membrane, resulting in the activation of AMPK and suppression of gluconeogenesis [142,143,144]. Recently, Ma et al. demonstrated that a clinically relevant low dose of metformin hampers the vacuolar H + -ATPase of the lysosomal proton pump (v-ATPase), a crucial macromolecular structure for AMPK activation after glucose starvation. Metformin, by binding PEN2, a subunit of γ-secretase, forms a complex with ATP6AP1, a subunit of v-ATPase, resulting in the inhibition of v-ATPase and activation of AMPK without causing an impact on cellular AMP levels [145]

Metformin has been shown to have synergistic activity with several anticancer drugs and facilitates the reduction in chemo and/or radioresistance in different types of tumors. In vitro and in vivo studies, performed on different tumor cells, show antiproliferative effects linked to different molecular mechanisms. In this regard, several clinical observations, carried out in recent years, show that metformin seems to be able to reduce the risk of cancer development in diabetic patients and improve the effectiveness of certain therapies and the survival time in patients with certain types of cancer, such as non-small cell lung cancer [146,147,148,149], pancreatic cancer [150,151], gastric cancer [152], prostate cancer [153,154], colorectal cancer [153,155], and breast cancer [156,157]. These data, although still awaiting further validation, support the possibility of using metformin as an adjuvant therapy against cancer development and progression [158,159,160].

#### Metformin as an Immunomodulator

Recently, there has been a great deal of attention paid to the actions of metformin on the immune system (see Box 1), and due to the close relationship between the immune system, the microbiota, and the metabolic rate [161,162,163,164], the effects of metformin on each one of these elements could contribute to its therapeutic effects and provide the rationale for new applications to ameliorate immunosenescence and aging.However, although several beneficial functions of metformin have been observed in multiple cellular processes, its contribution to immunomodulation is still poorly understood [165,166,167,168,169].

In an attempt to better investigate the effects, some simple in vivo models have been used, such as *Caenorhabditis elegans*, an appreciated genetic model for immune response. Using this model, various research groups have discovered several ortholog signaling pathways that play an important role in the control of innate immunity, such as the PMK-1/p38 MAPK pathway [170,171], the protein kinase D DKF-2 [172], the DAF-2/DAF-16 pathway [173], the protein-coupled receptor FSHR-1 [174], the MPK-1/ERK MAPK pathway [175], and the G protein GqαEGL-30 [176]. In a genetic screening performed on *C. elegans*, it was also hypothesized that metformin has healthy action through an H3K4 methyltransferase/demethylase complex, downregulating some targets, including mTOR and S6 kinase [177].Taken together, these results uncovered an alternative antibiotic-like mechanism through which metformin could benefit the immune response. Further supporting this hypothesis, the immunomodulatory properties of some antibiotics improve the long-term prognosis of patients with some chronic diseases [178,179]. For example, azithromycin, a macrolide, appears to have beneficial effects on the health of patients with chronic obstructive pulmonary disease and cystic fibrosis (CF) [180,181].

Additionally, proving its use as immunomodulator, metformin has been shown to increase the number and function of tumor-infiltrating lymphocytes (TILs) [182]. In this regard, in a study by Pereira et al., strong immunomodulatory effects of metformin are reported and it seems to also contribute to the reduction in lung metastases of melanoma cells [183]. Moreover, Cha et al. have shown that metformin reduces the stability and localization of the membrane of the programmed death-ligand 1 (PD-L1) and contributes to the improvement of the cytotoxic activity of T lymphocytes (CTL) against cancer cells [184]. Finally, metformin also exerts anti-inflammatory effects [185,186], and it has been reported that these effects could be related to the alteration of the intestinal microbiota [187].

Regarding the molecular mechanism involved, current data indicate that the immunomodulatory activity of metformin is linked to its direct effect on the cellular functions of various types of immune cells with the consequent induction of AMPK and subsequent inhibition of mTORC1 and inhibition of the production of mitochondrial ROS. Among the key immune events affected, it has been reported that the differentiation of monocytes into macrophages hampered inflammatory capacity. Furthermore, metformin treatment increases the differentiation of naïve T cells into regulatory T cells and memory T cells, decreasing the ability of neutrophils to initiate in NETosis. For this reason, thanks to this inhibitory effect on the activity of proinflammatory immune cells, metformin appears to have a beneficial role in reducing the burden of autoimmune diseases in several animal models and in some human studies [122]. Indeed, metformin has been used to upregulate AMPK and influence the development of memory T cells [182,188,189]. Indeed, metformin has been used as a tool to upregulate AMPK and demonstrate the importance of this energy sensor for the development of memory T cells [190]. In addition, the anti-inflammatory effects observed in multiple sclerosis [191] improve disease outcomes [192], suggesting that metformin may affect CD4 T-cell metabolism and reduce autoimmunity. Finally, the mechanism involved in the reduction in autoimmunity seems to be associated with the activation of AMPK, resulting from the inhibition of oxidative phosphorylation in B lymphocytes [193].

Importantly, metformin has also been observed to suppress inflammation associated with senescence [194] and, according to some in vivo and in vitro studies, to suppress cancer-related inflammation [195]. Furthermore, cancer immunology studies provide examples of enhanced immune function after the administration of metformin [182,196], while other studies highlight anti-inflammatory actions [191,194,195,197]. These data show the impaired activation of NF-κB, with a concomitant reduction in inflammatory cytokine secretion, although the molecular target of metformin leading to inhibition of NF-κB has not yet been identified [197]. In this regard, there is evidence that metformin inhibits ROS production in senescent cells [198], where mitochondrial ROS has been implicated in NF-κB activation [199]. Interestingly, metformin inhibits IKKα kinase but not p38 MAPK and since these two pathways are the downstream of TAK1, the drug is hypothesized to only interfere with the IKK/NF-κB pathway [194]. These anti-inflammatory and immunomodulatory effects may lead to new clinical applications for immunosenescence and aging [200].

Studies are ongoing to better elucidate the molecular pathways involved and to more precisely measure the expression of surface transporter proteins on effector and suppressor immune cells (such as the organic cation transporter 1, OCT-1) required for the import of metformin [201,202].

## 4. Conclusions

Immunosenescence is a gradual deterioration of the immune system that occurs physiologically with aging or due to pathological conditions. Potential natural and synthetic compounds to counteract this phenomenon are being studied, and pharmacological approaches include the use of several molecules, for example, growth factors, checkpoint inhibitors, mTOR/IGF-1 inhibitors, and others. Resveratrol, rapamycin, and metformin have shown efficacy in treating conditions related to immunosenescence and aging and their similarities in the mechanism of action have been described in this review. Additional studies aimed at better employing these compounds are needed, together with pharmacological strategies and dietary nutrients, particularly rich in bioactive components that can help to promote physiological immune functions and reduce the inflammatory status, ultimately maintaining a proper immune response during aging.

## Data Availability

Data sharing not applicable.
